# Depression and determinants among diabetes mellitus patients in Ethiopia, a systematic review and meta-analysis

**DOI:** 10.1186/s12888-023-04655-6

**Published:** 2023-03-29

**Authors:** Kirubel Dagnaw Tegegne, Natnael Atnafu Gebeyehu, Mesfin Wudu Kassaw

**Affiliations:** 1 Department of Nursing, College of Medicine and Health Science, Wollo University, Dessie, Ethiopia; 2 Department of Midwifery, College of Medicine and Health Science, Wolaita Sodo University, Wolita Sodo, Ethiopia; 3 School of Nursing, College of Health Science, Woldia University, Woldia, Ethiopia

**Keywords:** Depression, Determinants, Meta-analysis, Diabetes mellitus, Prevalence, Ethiopia

## Abstract

**Introduction:**

Primary studies have estimated the prevalence of depression and its determinants among diabetes patients. However, studies synthesizing this primary evidence are limited. Hence, this systematic review aimed to determine the prevalence of depression and identify determinants of depression among diabetes in Ethiopia.

**Methods:**

This systematic review and meta-analysis included a search of PubMed, Google Scholar, Scopus, Science Direct, PsycINFO, and Cochrane library. Data were extracted using Microsoft Excel and analyzed using STATA statistical software (v. 14). Data were pooled using a random-effects model. Forest plots, and Egger’s regression test were all used to check for publication bias. Heterogeneity *(I)*^*2*^ was computed. Subgroup analysis was done by region, publication year, and depression screening instrument. In addition, the pooled odds ratio for determinants was calculated.

**Results:**

Sixteen studies, including 5808 participants were analyzed. The prevalence of depression in diabetes was estimated to be 34.61% (95% CI: 27.31–41.91). According to subgroup analysis by study region, publication year, and screening instrument, the highest prevalence was observed in Addis Ababa (41.98%), studies published before 2020 (37.91%), and studies that used Hospital Anxiety and Depression Scale (HADS-D) (42.42%,) respectively. Older age > 50 years (AOR = 2.96; 95% CI: 1.71–5.11), being women (AOR = 2.31; 95% CI: 1.57, 3.4), longer duration with diabetes (above 5 years) (AOR = 1.98; 95% CI: 1.03–3.8), and limited social support (AOR = 2.37; 95% CI: 1.68–3.34), were the determinants of depression in diabetic patients.

**Conclusion:**

The results of this study suggest that the prevalence of depression in diabetes is substantial. This result underscores the importance of paying particular attention to prevent depression among diabetes. Being older, not attending formal education, longer duration with diabetes, having comorbidity, and low adherence to diabetes management were all associated. These variables may help clinicians identify patients at high risk of depression. Future studies focusing on the causal association between depression and diabetes are highly recommended.

**Supplementary Information:**

The online version contains supplementary material available at 10.1186/s12888-023-04655-6.

## Background

Depression refers to persistent sadness and a loss of interest or pleasure in a previously enjoyable activities[1]. Diabetes, characterized by high blood sugar levels, is a common chronic condition with a rapid surge in prevalence rate. According the International Diabetes Federation (IDF) estimates in 2017, the number of people living with diabetes aged 20–79 years was 424.9 million and this is projected to rise to 629 million[2]. Diabetes prevalence, deaths from diabetes, and related healthcare expenditures impose a significant burden in social, financial, and health system worldwide. In Sub-Saharan countries, diabetes resulted crisis in the health care system and the economy, with the five leading countries with diabetes in 2017 being Ethiopia, South Africa, the Democratic Republic of the Congo, Nigeria, and Tanzania[3].

The effects of diabetes extend from short-term complications (e.g., hypoglycemia) and long-term complications (e.g., cardiovascular disease, neuropathy, nephropathy, and retinopathy), to a serious physical and mental health impairments[4, 5].

Studies have shown that diabetes is frequently associated with mental health disorders such as depression and anxiety[6, 7]. The risk of developing depression in diabetes is due to the psychological stress following diagnoses of diabetes, altered glucose transport from hyperglycemia, and treatment of the disease itself [8, 9]. The prevalence of depression in diabetes is much higher compared to the general population, approximately three times higher in patients with type 1 diabetes, and twice higher in type 2 diabetes (T2DMA)[10]. Two meta-analyses reported that people with diabetes are 15–24% more likely to develop depression compared to people without diabetes[11, 12]. In Africa, a study by Ogunsakin R. et al. reported that depression in patients with diabetes is 40%[13]. A recent review in Ethiopia also revealed that the prevalence of depression in diabetes patients is 39.73%[14]. Depressed individuals with diabetes report lower quality of life [15] have higher HbA1c levels, indicating suboptimal glycemic control [16] and are characterized by poor self-care behavior that may contribute to suboptimal glycemic control [17]. Diabetes patients demonstrate lower levels of physical activity [18], have more negative appraisals of insulin therapy [19], are likely to be non-adherent to treatment regimen and have unhealthy eating behaviors[17].

Despite multiple cross-sectional surveys have investigated the prevalence of depression among diabetes patients in Ethiopia, the results vary. In addition, several studies that reported diverse determinants associated with the prevalence of depression in patients, such as gender, age, complications, lifestyle, and social support remained inconsistent [20–22]. The last reported meta-analysis that provided a prevalence of depression among diabetes in Ethiopia was in 2018, and since then a considerable number of primary studies have been published. Additionally, the previous meta-analysis did not include analysis of factors of depression. Studies revealed that diabetes are at higher risk for depression due to nature of illness, long-term diseases management and complications [23–25]. Thus, systematic review and meta-analysis are needed to highlight the overall picture of depression in diabetes nationally. Estimating the country-level burden of depression allows the way to design strategies and policies on health education, screening, and early intervention of depression. Therefore, this systematic review and meta-analysis aimed to evaluate the prevalence of depression and its determinants among diabetes in Ethiopia.

## Methods

This meta-analysis was conducted following the Preferred Reporting Items for Systematic reviews and Meta-Analyses (PRISMA) guidelines[26] for reporting (Supplementary Material 1). We registered this meta-analysis with PROSPERO (CRD42022355229) to avoid unnecessary replication of this project.

### Search strategy

An exhaustive literature search in the Pub Med, Science Direct, Scopus, Cochrane library and Google Scholar, PsyINFO databases was carried out. The search was done using the following keywords combined with each other: ((((((((((((((((((prevalence) OR (magnitude)) OR (epidemiology)) AND (Depression)) OR (depressive disorder)) OR (depressive symptoms)) OR (major depression)) OR (mental health problems)) OR (distress)) OR (psychological distress)) AND (diabetes mellitus)) OR (diabetes)) OR (DM)) AND (determinants)) OR (factors)) OR (associated factors)) OR (predictors)) AND (adults)) AND (Ethiopia). Search terms were based on PICO principles to retrieve relevant articles through the databases mentioned above. The search period was from June 1 /2022, to July 30/2022.

### Eligibility criteria

This meta-analysis included studies that reported the prevalence of depression in adults (18 years and above), only English language publications, both published and unpublished studies with full text, and studies conducted in Ethiopia. No restrictions were made on the studies publication year. Articles with no accessibility to the full text, language other than English, publication, and articles other than original research, i.e., symposium or conference abstracts, book chapters, review papers, and case reports were excluded.

### Quality assessment

Two authors (KDT and NAG) independently examined the included studies using the Joanna Briggs Institute (JBI) checklist for prevalence studies[27]. Any disagreements arose during the process were resolved by a discussion led by the third author (MWK) and an agreement was reached. The critical analysis checklist has eight parameters with yes, no, unclear, and not applicable responses. The checklist involve the following questions: Q1: Were the criteria for inclusion in the sample clearly defined?, Q2: Were the study subjects and the setting described in detail?, Q3: Was the exposure measured validly and reliably?, Q4: Were the standard criteria measured the outcome objectively?, Q5 Were confounding factors identified?, Q6: Were strategies to deal with confounding factors stated?, Q7: Were the outcomes measured validly and reliably?, and Q8: Was appropriate statistical analysis used?. Studies were considered low risk when their score is less than 50% and above on the quality assessment indicators, as reported in a supplementary file (Supplementary Material 2).

### Risk of bias assessment

Risk of bias was assessed by two authors (KDT and NAG) independently using the bias assessment tool developed by Hoy et al.[28], consisting of ten items that evaluate four domains of bias, internal and external validity. Disagreements raised during the assessment were resolved by discussion led by a third author (MWK) and finally consensus was reached. The first four items (items1– 4) evaluate the presence of selection bias, non-response bias, and external validity. The remaining six items (items 5– 10) assess the presence of biases in measurement and analysis, and internal validity. Studies that got “yes” answers to eight or more of the ten questions were labeled as having “low risk of bias.“ Studies with a “yes” response for six to seven out of ten questions would be considered “moderate risk,“ while those with a “yes” response for five or fewer out of ten questions would be considered “high risk.“ as reported in a supplementary file (Supplementary Material 3).

### Data extraction

Microsoft Excel spreadsheet (2016) and STATA version 14 software were utilized for data extraction and analysis, respectively. All relevant data were extracted using a standardized Joanna Briggs Institute data extraction format by two authors (KDT and NAG). Presence of any disagreements raised during data extraction were resolved by a third author (MWK) and agreement was reached. The data automation tool was not used due to this study’s absence of the paper form (manual data). Author names, publication year, study region, study design, age of participants, the prevalence of depression, depression screening instrument, and sample size of each paper was extracted.

### Data analysis

After all relevant findings extracted, the data were exported from excel into STATA 14 for analysis. Pooled prevalence with corresponding 95% CIs was calculated. The meta-analyses results were displayed in forest plots and tables. Publication bias was checked by visual inspecting the funnel plot and objectively by Egger’s regression tests, [29] and *p*-value less than 0.05 indicates a statistical significance[30]. Statistical heterogeneity was examined using the *I*^2^. Based on the Cochrane Collaboration recommendations, *I*^2^ values represented moderate (30-60%), substantial (50-90%), and considerable (75-100%) heterogeneity[31]. Subgroup analysis were conducted according to study region and publication year (before 2020 and, at 2020 and after) and depression screening instrument. Sensitivity analyses were also carried out to examine the effect of omitting a single study on the overall prevalence.

## Results

### Study selection

**Fig. 1:**
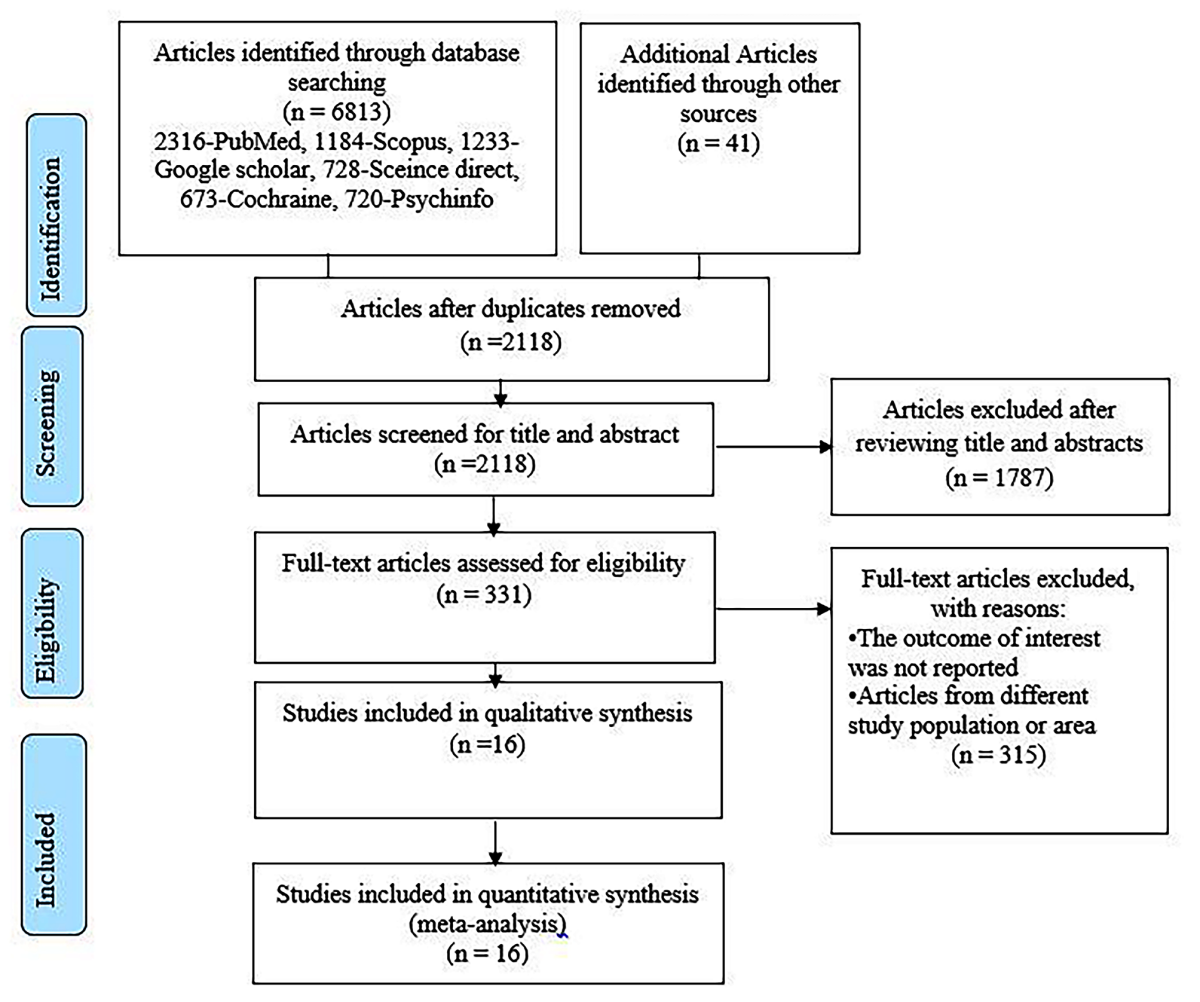
PRISMA flow chart illustrating the process of search and selection of studies included in the present systematic review and meta-analysis

The literature search has yielded 6854 records through electronic databases of PubMed, Scopus, Google Scholar, Science direct, Cochrane library, and Psycinfo. After duplicates were removed, 2118 articles remained. Then, 1787 studies were excluded after reviewing for full title and abstracts from the remaining 2118 studies. Therefore, 331 full-text studies were assessed for eligibility criteria, which further excluded 315 studies due to unreported outcome of interest and studies from different study population or area. Finally, 16 articles were included as criteria for this systematic review and meta-analysis study (Fig. 1).

### Studies characteristics

The included studies contained 5808 adult subjects. The studies were published between 2013 and 2021. All studies were institutional cross-sectional in nature. Five studies conducted in Oromia region [22, 32–35], three in Addis Ababa [21, 36, 37], three in Amhara[20, 38, 39], three in SNNP[40–42], one in Tigrai [43] and one in Harari[44]. The sample sizes ranged from 260 to 423. Nine studies assessed depression using the 9-item Patient Health Questionnaire (PHQ-9)[45], 3 used the Beck Depression Inventory (BDI)[46], 2 used the Hospital Anxiety and Depression Scale (HADS-D)[47], and 2 used other methods[48, 49]. The prevalence of depression ranges from 9.19 to 60.06. All studies were assessed by using Joanna Briggs Institute (JBI) quality appraisal checklist. Therefore, sixteen studies were evaluated, and all received a quality score of 75% or above on the quality scale, indicating that they are low risk and included in the analysis (Table 1).

**Table 1:** Summary characteristics of studies included in the meta-analysis

Author	Year	Region	Setting	Study design	Instrument used	Sample size	Response rate (%)	Prevalence	Quality
Adane A. et al.	2020	SNNP	Institution-based	Cross-sectional	PHQ-9	398	94	36.93	Low-risk
Anteneh M. et al.	2016	Amhara	Institution-based	Cross-sectional	PHQ-9	415	98.3	15.42	Low-risk
Bereket B. et al.	2020	SNNP	Institution-based	Cross-sectional	PHQ-9	260	96.3	41.54	Low-risk
Biruk S. et al.	2020	Oromia	Institution-based	Cross-sectional	Kessler 6 scale	359	88.4	9.19	Moderate-risk
Bonsa A. et al.	2021	Oromia	Institution-based	Cross-sectional	BDI	321	90.7	34.89	Low-risk
Mengistu E. et al.	2013	Addis Ababa	Institution-based	Cross-sectional	Hamillton depression rating scale	313	Not reported	60.06	Low-risk
Gedion A. et al.	2020	SNNP	Institution-based	Cross-sectional	PHQ-9	418	98.1	28.71	Low-risk
Mogessie N. et al.	2020	Amhara	Institution-based	Cross-sectional	PHQ-9	421	96.3	38.72	Low-risk
Mohammedamin H. et al.	2020	Oromia	Institution-based	Cross-sectional	HADS-D	397	Not reported	37.78	Low-risk
Mohammed E. et al.	2021	Harari	Institution-based	Cross-sectional	PHQ-9	401	98	48.88	Low-risk
Nigus A. et al.	2020	Addis Ababa	Institution-based	Cross-sectional	PHQ-9	403	99	21.34	Low-risk
Sisay D. et al.	2014	Oromia	Institution-based	Cross-sectional	BDI	335	96	43.58	Low-risk
Tesfa D. et al.	2016	Addis Ababa	Institution-based	Cross-sectional	PHQ-9	264	95.6	44.7	Low-risk
Teshager W. et al.	2020	Amhara	Institution-based	Cross-sectional	PHQ-9	416	100	29.33	Low-risk
Tilahun B. et al.	2017	Tigrai	Institution-based	Cross-sectional	BDI	264	Not reported	17.05	Moderate-risk
Tiki/2017	2021	Oromia	Institution-based	Cross-sectional	HADS-D	423	100	47.04	Low-risk

**Note**: BDI- Beck Depression Inventory, HADS- D **–**Hospital Anxiety and Depression Scale, PHQ-9 – Patient Health Questionnaire

## Meta-analysis

### Prevalence of depression among diabetes in Ethiopia

**Fig. 2:**
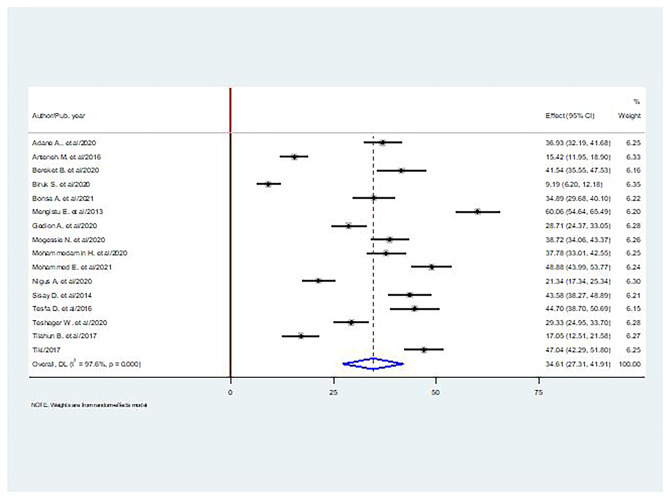
The pooled prevalence of depression among diabetes in Ethiopia

Sixteen studies involving 5808 patients with diabetes reported the prevalence of depression. The random-effects pooled prevalence of depression among diabetes in Ethiopia was 34.61% (95% CI: 27.31–41.91), with significant heterogeneity observed between studies (I^2^ = 97.6%, p < 0.000). The studies were conducted on diabetes patients 18 years old and older (Fig. 2).

### Sub-group analysis

We observed high heterogeneity between studies (I^2^ = 97.6%). As a result, sub-group analysis is conducted based on study region, publication year and diagnosis method. As per the region, the highest pooled prevalence of depression in diabetes was observed at Addis Ababa, 41.98%, (95% CI, 17.88–66.07, I^2^ = 98.5%) and the lowest was in Amhara, 27.75%, (95% CI, 13.99–41.52, I^2^ = 97.0%). In the context of publication year, the pooled prevalence appear to be higher in studies done before 2020, which is 37.91%, (95% CI, 22.71–53.11, I^2^ = 98.4%). Regarding the screening instrument used, higher prevalence estimates were found among studies that used HADS-D 42.42%, (95% CI 33.34–51.49, I^2^ = 86.2%)and the lowest was observed among studies that used BDI, 31.79%, 95% CI 15.97–47.61, I^2^ = 96.7%). (Table 2).

**Table 2:** Sub-group analysis of studies included in the meta-analysis on the prevalence and determinants of depression among diabetes in Ethiopia

Sub-group	Random effects(95% CI)	Test of heterogeneity (I^2^) (%)
**By region**		
Amhara	27.75% (13.99–41.52%)	97.0
Oromia	34.44% (18.11–50.77%)	98.5
Addis Ababa	41.98% (17.88–66.07%)	98.5
SNNP	35.52% (28.14–42.89%)	84.8
Other	32.95% (1.75–64.14%)	98.9
**Publication year**		
Before 2020	37.91% (22.71–53.11%)	98.4
2020 and after	32.65% (24.44–40.85%)	97.1
**By screening instrument**		
PHQ-9	33.83% (26.2-41.46%)	96.0
BDI	31.79% (15.97–47.61%)	96.7
HADS-D	42.42% (33.34–51.49%)	86.2
Other	34.58% (15.28–84.43%)	99.6

## Meta regression

To identify the sources of heterogeneity meta-regression was performed using study year and sample size as a covariate. Thus, it was indicated that there is no effect of year of study and sample size on heterogeneity between studies as indicated by insignificant p-value (Table 3).

**Table 3:** Meta-regression analysis of factors affecting between-study heterogeneity

Heterogeneity source	Coefficients	Std. Err.	P-value
Sample size	0.00046	0.0112	0.968
Study year	-0.0433	0.2628	0.872

### Publication bias

**Fig. 3:**
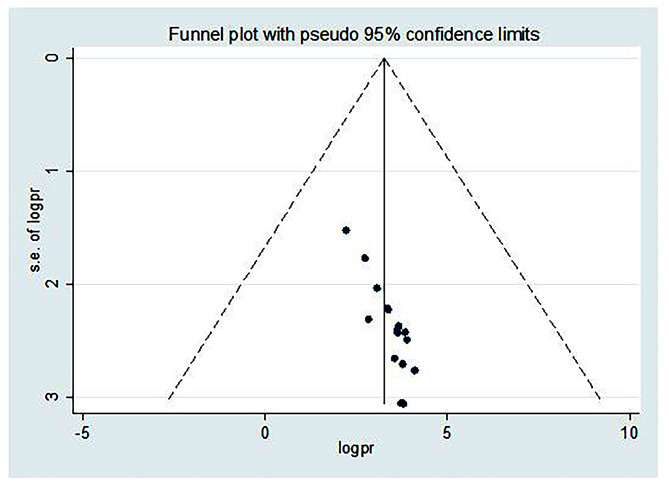
Funnel plot which shows the asymmetrical distribution of studies

The presence of publication bias was checked using funnel plot visualization and more objectively by Egger’s test (P < 0.05). The funnel plot showed an asymmetrical distribution that indicated a possibility of publication bias (Fig. 3). This was supported by the results of Egger test, which indicated a possibility of publication bias (P = 0.000). To reduce this publication bias, Trim and fill analysis was conducted and the result indicated the presence of 2 unpublished studies. (Supplementary Material 4).

#### Sensitivity analysis for the studies included

**Fig. 4:**
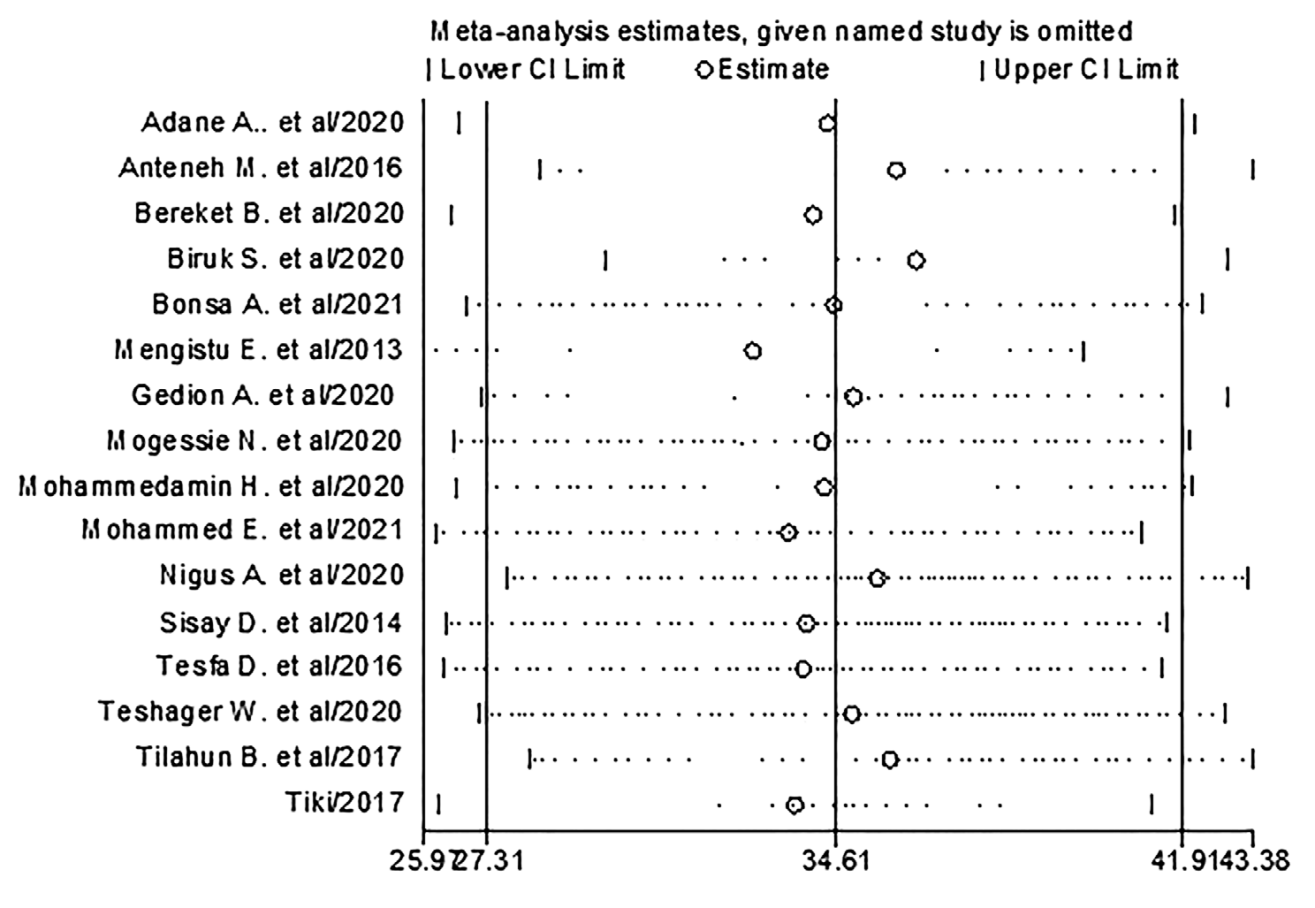
Results of sensitivity analysis of the 16 studies in the meta-analysis of depression in diabetes

To check the individual effect of included studies on the pooled prevalence of depression among diabetes in Ethiopia, sensitivity analysis was performed using random effect model. The findings revealed that there was no strong evidence that a single study had an impact on the overall result of the meta-analysis, since the single study estimates were closer to the combined estimate. Leaving out one study at a time, the pooled estimated prevalence of depression is ranged from 32.91 (95% CI, 25.97–39.85) to 36.31 (95%, 29.78–42.84). In a leave-1-out analysis, omitting the study of Ebrahim et al. [44] reduced the prevalence to 33.65% (95% CI, 26.26–41.05). Similarly, removing the study of Habtewold et al[21] lowered the prevalence of depression to 33.94%(95% CI, 26.4-41.49). Conversely, removal of Tusa et al[35] increased the prevalence to 36.31% (95% CI, 29.78–42.84) (Fig. 4).

### Factors associated with depression among diabetes

In this systematic review and meta-analysis we identified a variety of factors associated with depression in diabetes. As a result, age older than 50 years, being female, longer duration with diabetes (> 5 years), limited social support, are the determinants for depression among diabetes, whereas diabetic complications was not a determinant for depression among diabetes.

### Association of older age with depression

We examined numerous study to assess the association of age with depression in diabetic patients. Accordingly, older age (> 50 years) was reported to be a determinant for depression by three primary studies included in this review [20, 33, 39]. A total of 1158 subjects were included to analyze the association of older age with depression. The pooled odds ratio showed that older age, > 50 years were nearly 3 times more likely to have depression than younger ages (AOR = 2.96; 95% CI: 1.71, 5.11), I^2^ = 0.00%, P < 0.837) (Table 4).

### Association of sex and depression

To examine the association between sex and depression in diabetic patients, we reviewed all studies included in this systematic review. Being women was identified as a significant factor associated with depression in four primary studies included in this meta-analysis [20, 22, 34, 44]. A total of 1637 subjects were included to analyze the association between and being women and depression. The odds of depression among women were 2.31 times higher than educated (AOR = 2.31; 95% CI: 1.57, 3.4), I^2^ = 62.1%, P = 0.048) (Table 4).

### Association of longer duration of diabetes with depression

Five primary studies that evaluated longer duration of diabetes in relation to depression in diabetes were included [50–55]. This meta-analysis included a sample of 2034 individuals. The pooled odds ratio showed that longer duration of diabetes (> 5years) nearly 2 times more likely to be depressed than their counterparts (AOR = 1.98; 95% CI: 1.03–3.8), I^2^ = 85.1%, P = 0.000) (Table 4).

### Association of social support with depression

Eight primary studies with a total subject of 2883 reported limited social support as a determinant for depression in diabetic patients [20–22, 33, 36, 39, 42, 44]. The pooled odds ratio showed that diabetes peoples with limited social support had a 2.37 times higher risk to experience depression as compared to diabetes having good social support (AOR = 2.37; 95% CI: 1.68–3.34), I^2^ = 58.8%, P = 0.017) (Table 4).

### Association of diabetic complications and depression

Four primary studies with a total subject of 1367 reported assessed the association between having diabetic complications with depression in diabetic patients [21, 33–35]. The pooled odds ratio showed that having diabetic complications was not found to be a determinant for depression in diabetic patients (AOR = 1.92; 95% CI: 0.78–4.72), I^2^ = 69.4%, P = 0.02) (Table 4).

**Table 4:** Summary estimates of odds ratio for determinants of depression in diabetes

Determinants	Number of studies	Total sample (n)	Pooled OR (95% CI)	Heterogeneity I^2^ (%)
Older age	3	1158	2.96 (1.71, 5.11)^*^	0.00
Female sex	4	1637	2.31 (1.57–3.4)^*^	62.1
Longer duration of diabetes	5	2034	1.98 (1.03–3.8)^*^	85.1
Poor social support	8	2883	2.37 (1.68–3.34)^*^	58.8
Diabetic complications	4	1367	1.92 (0.78–4.72)	69.4
Note: ^*^ indicates significant variables.				

## Discussion

This review was conducted to determine the pooled prevalence and factors of depression among diabetes aged 18 years and older in Ethiopia. This study presented a meta-analysis of 16 articles with a total of 5808 study subjects. This meta-analysis indicated that a pooled prevalence of depression among diabetes is 34.61% (95% CI: 27.31–41.91). The findings of this study implies diabetes peoples are as a high-risk group for depression. This underscores the value of designing regular depression screening and management programs in diabetes.

The prevalence rate in the present meta-analysis was comparable with a previous systematic reviews and meta-analyses conducted among diabetic populations in Ethiopia[14]. Similarly, Consistent with our result, a recent meta-analysis in Africa reported a 40% prevalence of depression in diabetes[13]. Our study confirmed previous findings of the higher prevalence of depression among diabetes in Saudi Arabia[56]. Conversely, our prevalence result is lower than a study reported in Poland, 51%[25]. The relative lower depression prevalence observed in Ethiopia might be attributed to differences in depression screening methods used between the two studies. In addition, In Ethiopia, a culture of good social support and social integration through during different social events could reduce the occurrence of depression in diabetes. Studies reported that peoples with good social support are at lower risk for depression compared to those without good social support[57].

A wide range of depression prevalence was found among the included studies, which could be attributable to the high heterogeneity of the samples included in this review. As a result, we further conducted subgroup analyses by study region, study year and diagnosis methods used. Thus, based on region, Addis Ababa has the highest prevalence of depression, 41.98%. The higher reported prevalence of depression among diabetes in urban towns like Addis Ababa, is also supported by a previous meta-analysis in Ethiopia[14]. Areas with high population densities are characterized by higher rates of criminality, mortality, social isolation, air pollution and noise[58]. As the extent of these social problems is related to urbanization it is often assumed that rates of psychiatric disorders, including depression are also correlated with urbanization.

Regarding on subgroup analysis based on study year, we found a higher prevalence of depression among studies published before 2020 which is 37.91%, and 32.65% in studies published at 2020 and latter. According to subgroup analysis by instruments methods used to measure depression, a higher prevalence was reported by studies that used HADS-D, 42.42%, and the lower prevalence of depression was observed in studies that used BDI, 31.79%. A consistent depression scale should be widely used to promote standardization and comparability across studies. The heterogeneity of studies while reporting the prevalence estimates ranges from 96 to 99.6%. The use of different screening methods for depression, variation in the sample size and some other unknown factors among individual studies might contributed for the considerable heterogeneity in this meta-analysis.

Our finding showed that age older than 50 years, being female, longer duration with diabetes (> 5 years), limited social support, were the factors for depression among diabetes. Moreover, this review found that older age diabetic patients > 50 years were more likely to have depression. This result were consistent with previous studies[59, 60]. With the decline of health level and prolonged duration of diabetes, elderly patients with diabetes are more likely to suffer from multiple diseases and complications, which may contribute to a high risk of depression. This implies that the importance of strengthening depression screening and interventions activities for older diabetes.The pooled odds of depression among female DM patients were 2.31 times higher than those male DM patients.

Many epidemiological studies pointed out the gender gap in the prevalence of depression, that female are more likely to suffer from depression than male during their lifetime[61, 62], which may be due to environmental, hormonal, genetic factors [63]. The above factors might lead to the observed gender differences between women and men as reported in our and other relevant meta-analysis [64–67].

Our study demonstrated that patients with a duration of diabetes more than 5 years were at higher odds to develop depression. This result were consistent with findings in Qatar[68], US[69], and Australia[70]. Previous studies revealed that long duration of diabetes was associated with a high risk of micro vascular diseases[71, 72] such as nephropathy, retinopathy, neuropathy, and macro vascular complications which could be linked to depression[73]. Meanwhile, the duration of diabetes varies inversely with quality of life, particularly in patients with more than 10 years[74]. Therefore, diabetes patients with longer duration should be classified as a risk group for developing depression, requires more careful and different assessment and treatment strategies to prevent its occurrence.

Poor social support is the other critical factor associated with depression in diabetes. Our study indicated that diabetes with poor social support were 2.37 times more likely to have depression compared to good social support. Poor social support is the primary factor that causes patients with diabetes to experience depression, according to a prior meta-analysis[57, 75]. Additionally, a study by Ioannou et al. emphasized the importance of social support in preventing depression[76]. Social support makes people feel valued and connected to their social networks, which enhances mental health and quality of life. This sense of belonging is linked to better mental healthoutcomes, making it a method of preventing depression[77]. Interventions based on peer and family support can be integrated to diabetes self-management[78]. Technology is a promising way to provide a family and friend social support. For example, by utilizing an online health community social support and consultation from doctors can be delivered. Overall, the implications for policy makers, health professionals and supporting agencies are to encouraging screening of diabetes for depression, integrating mental health therapies alongside with the usual diabetes care and improving overall health outcome.

## Conclusion

In summary, our study demonstrates that the prevalence of depression in diabetes is higher. Factors such as older age, being women, longer duration of diabetes, and low social support were found to be the factors for depression. The increased risk of depression in diabetic patients highlights the importance of integrating the evaluation and treatment of depression with diabetes management in healthcare settings. To that end, it is vital that healthcare professionals pay special attention to specific groups, such as older diabetics, women with diabetes, diabetes with longer duration, and those with low social support to prevent from depression among diabetic patients. Future studies are recommended to better understand the real burden and identify all variables associated with depression in diabetes.

### Strength and limitations

This study has several strengths. It provides an up-to-date literature review, which includes both the prevalence of depression and determinants among diabetic patients. It provides evidence that can be used as a reference point for future research focusing on specific diabetic sub-populations. However, there are also several limitations we need to consider. First, articles were restricted to only being published in the English language, which may result in the exclusion of other articles. Second, there was a marked heterogeneity among the included studies.

## Electronic supplementary material

Below is the link to the electronic supplementary material.

Supplementary Material 1 Table: PRISMA 2020 Checklist

Supplementary Material 2 Table: Quality assessment for the included Studies

Supplementary Material 3 Table: Risk of bias of assessment for the cross-sectional studies

Supplementary Material 4 Funnel plot to show trim and fill analysis

Supplementary Material 5 Legends

## Data Availability

All relevant data are within the Manuscript and its Supporting Information files.

## Abbreviations

DM: Diabetes Mellitus
HgA1C: Glycated Hemoglobin
IDF: International Diabetes Federation
JBI: Joanna Briggs Institute
PRISMA: Preferred Reporting Items for Systematic Reviews and Meta-Analyses

## References

[1] Organization WH. Health topics: depression. Geneva, Switzerland: World. 2014.

[2] Carracher AM, Marathe PH, Close KL. International diabetes federation 2017. Wiley Online Library; 2018. 10.1111/1753-0407.12644 29345068

[3] Peer N, Kengne A-P, Motala AA, Mbanya JC (2014). Diabetes in the Africa Region: an update. Diabetes Res Clin Pract.

[4] Alzoubi A, Abunaser R, Khassawneh A, Alfaqih M, Khasawneh A, Abdo N (2018). The bidirectional relationship between diabetes and depression: a literature review. Korean J family Med.

[5] Kalra S, Jena BN, Yeravdekar R (2018). Emotional and psychological needs of people with diabetes. Indian J Endocrinol Metabol.

[6] Andreoulakis E, Hyphantis T, Kandylis D, Iacovides A (2012). Depression in diabetes mellitus: a comprehensive review. Hippokratia.

[7] Sharma K, Dhungana G, Adhikari S, Bista Pandey A, Sharma M. Depression and anxiety among patients with type ii diabetes mellitus in Chitwan Medical College Teaching Hospital, Nepal. Nursing research and practice. 2021;2021. 10.1155/2021/8846915 PMC7817292 33520315

[8] Anderson RJ, Freedland KE, Clouse RE, Lustman PJ (2001). The prevalence of comorbid depression in adults with diabetes: a meta-analysis. Diabetes Care.

[9] Engum A (2007). The role of depression and anxiety in onset of diabetes in a large population-based study. J Psychosom Res.

[10] Roy T, Lloyd CE (2012). Epidemiology of depression and diabetes: a systematic review. J Affect Disord.

[11] Mezuk B, Eaton WW, Albrecht S, Golden SH (2008). Depression and type 2 diabetes over the lifespan: a meta-analysis. Diabetes Care.

[12] Nouwen A, Winkley K, Twisk J, Lloyd C, Peyrot M, Ismail K (2010). Type 2 diabetes mellitus as a risk factor for the onset of depression: a systematic review and meta-analysis. Diabetologia.

[13] Ogunsakin RE, Olugbara OO, Moyo S, Israel C (2021). Meta-analysis of studies on depression prevalence among diabetes mellitus patients in Africa. Heliyon.

[14] Teshome HM, Ayalew GD, Shiferaw FW, Leshargie CT, Boneya DJ. The prevalence of depression among diabetic patients in Ethiopia: a systematic review and meta-analysis, 2018. Depression research and treatment. 2018;2018. 10.1155/2018/6135460 PMC5989296 29951313

[15] Schram MT, Baan CA, Pouwer F (2009). Depression and quality of life in patients with diabetes: a systematic review from the european depression in diabetes (EDID) research consortium. Curr Diabetes Rev.

[16] Lustman PJ, Clouse RE (2005). Depression in diabetic patients: the relationship between mood and glycemic control. J Diabetes Complicat.

[17] Egede LE (2005). Effect of depression on self-management behaviors and health outcomes in adults with type 2 diabetes. Curr Diabetes Rev.

[18] Koopmans B, Pouwer F, de Bie RA, van Rooij ES, Leusink GL, Pop VJ (2009). Depressive symptoms are associated with physical inactivity in patients with type 2 diabetes. The DIAZOB Primary Care Diabetes study. Fam Pract.

[19] Makine C, Karşıdağ Ç, Kadıoğlu P, Ilkova H, Karşıdağ K, Skovlund S (2009). Symptoms of depression and diabetes-specific emotional distress are associated with a negative appraisal of insulin therapy in insulin‐naïve patients with type 2 diabetes mellitus. A study from the European Depression in Diabetes [EDID] Research Consortium. Diabet Med.

[20] Abate TW, Gedamu H (2020). Psychosocial and clinical factors associated with depression among individuals with diabetes in Bahir Dar City Administrative, Northwest Ethiopia. Ann Gen Psychiatry.

[21] Habtewold TD, Alemu SM, Haile YG (2016). Sociodemographic, clinical, and psychosocial factors associated with depression among type 2 diabetic outpatients in Black Lion General Specialized Hospital, Addis Ababa, Ethiopia: a cross-sectional study. BMC Psychiatry.

[22] Jarso MH, Likasa DD. Prevalence and associated factors of depression among diabetic outpatients in Ethiopia. The primary care companion for CNS disorders. 2020;22(2):26230. 10.4088/PCC.19m02479 32243106

[23] AbdElmageed RM, Hussein SMM. Risk of depression and suicide in diabetic patients.Cureus. 2022;14(1). 10.7759/cureus.20860 PMC8803388 35145767

[24] Aschner P, Gagliardino JJ, Ilkova H, Lavalle F, Ramachandran A, Mbanya JC (2021). High prevalence of depressive symptoms in patients with type 1 and type 2 diabetes in developing countries: results from the International Diabetes Management Practices Study. Diabetes Care.

[25] Florek-Luszczki M, Choina P, Kostrzewa-Zablocka E, Panasiuk L, Dziemidok P. Medical and socio-demographic determinants of depressive disorders in diabetic patients.Annals of Agricultural and Environmental Medicine. 2020;27(2). 10.26444/aaem/118529 32588602

[26] Moher D, Liberati A, Tetzlaff J, Altman DG, Group* P. Preferred reporting items for systematic reviews and meta-analyses: the PRISMA statement. Ann Intern Med. 2009;151(4):264–9. 10.7326/0003-4819-151-4-200908180-00135 19622511

[27] Institute JB (2017). JBI critical appraisal checklist for studies reporting prevalence data.

[28] Hoy D, Brooks P, Woolf A, Blyth F, March L, Bain C (2012). Assessing risk of bias in prevalence studies: modification of an existing tool and evidence of interrater agreement. J Clin Epidemiol.

[29] Egger M, Smith GD, Schneider M, Minder C (1997). Bias in meta-analysis detected by a simple, graphical test. BMJ.

[30] Sedgwick P. Meta-analyses: what is heterogeneity? Bmj. 2015;350. 10.1136/bmj.h1435 25778910

[31] Higgins J. Cochrane handbook for systematic reviews of interventions. In: Higgins JPT, Thomas J, Chandler J, Cumpston M, Li T, Page MJ, et al. editors. Cochrane Handbook for systematic reviews of interventions. Wiley; 2019. 10.1002/14651858.ED000142 PMC10284251 31643080

[32] Dejene S, Negash A, Tesfay K, Jobset A, Abera M (2014). Depression and diabetes in jimma university specialized hospital, Southwest Ethiopia. J Psychiatry.

[33] Geleta B, Dingata S, Emanu M, Kebede E, Eba L, Abera K (2021). Prevalence of depression and associated factors among type 2 diabetes patients attending hospitals in Ilu AbaBor and Bunno Bedelle Zones, South West Ethiopia, 2020: a cross sectional study. J Depress Anxiety.

[34] Tiki T (2017). Prevalence and associated factors of depression among type 2 diabetes mellitus patients on follow up at ambo general hospital, Oromia regional state, Ethiopia, institutional based cross sectional study. J Depress Anxiety.

[35] Tusa BS, Alemayehu M, Weldesenbet AB, Kebede SA, Dagne GA. Prevalence of depression and associated factors among diabetes patients in East shewa, Ethiopia: Bayesian approach. Depression research and treatment. 2020;2020. 10.1155/2020/4071575 PMC7596491 33145110

[36] Engidaw NA, Wubetu AD, Basha EA (2020). Prevalence of depression and its associated factors among patients with diabetes mellitus at Tirunesh-Beijing general hospital, Addis Ababa, Ethiopia. BMC Public Health.

[37] Erkie M, Feleke Y, Desalegne F, Anbessie J, Shibre T (2013). Magnitude, clinical and sociodemographic correlate of depression in diabetic patients, Addis Ababa, Ethiopia. Ethiop Med J.

[38] Birhanu AM, Alemu FM, Ashenafie TD, Balcha SA, Dachew BA. Depression in diabetic patients attending university of gondar hospital diabetic clinic, Northwest Ethiopia. Diabetes, Metabolic Syndrome and Obesity: Targets and Therapy. 2016;9:155. 10.2147/DMSO.S97623 PMC4869639 27274296

[39] Necho M, Tsehay M, Getachew Y (2020). Half of type 1 and nearly four in ten of type 2 diabetes patients were living with depression in North West Ethiopia, Amhara region. J Dep Anxiety.

[40] Asefa A, Zewudie A, Henok A, Mamo Y, Nigussie T. Depression and its associated factors among diabetes mellitus patients attending selected hospitals in Southwest Ethiopia: a cross-sectional study. Psychiatry journal. 2020;2020. 10.1155/2020/6486030 PMC7174965 32328503

[41] Azeze GA, Adema BG, Adella GA, Demissie BW, Obsa MS. Factors associated with untreated depression among type 2 diabetic patients at Halaba Kulito Hospital, South Ethiopia: a cross-sectional study. Diabetes, Metabolic Syndrome and Obesity: Targets and Therapy. 2020;13:2189. 10.2147/DMSO.S255360 PMC7323570 32612374

[42] Gebre BB, Anand S, Assefa ZM. Depression and its predictors among diabetes mellitus patients attending treatment in Hawassa university comprehensive specialized Hospital, southern Ethiopia. Journal of Diabetes Research. 2020;2020. 10.1155/2020/7138513 PMC7204154 32405504

[43] Mossie TB, Berhe GH, Kahsay GH, Tareke M (2017). Prevalence of depression and associated factors among diabetic patients at Mekelle City, North Ethiopia. Indian J Psychol Med.

[44] Ebrahim M, Tamiru D, Hawulte B, Misgana T (2021). Prevalence and associated factors of depression among diabetic outpatients attending diabetic clinic at public hospitals in Eastern Ethiopia: a cross-sectional study. SAGE open medicine.

[45] Kroenke K, Spitzer RL, Williams JB (2001). The PHQ-9: validity of a brief depression severity measure. J Gen Intern Med.

[46] Beck AT, Ward CH, Mendelson M, Mock J, Erbaugh J (1961). An inventory for measuring depression. Arch Gen Psychiatry.

[47] Zigmond AS, Snaith RP (1983). The hospital anxiety and depression scale. Acta psychiatrica Scandinavica.

[48] Kessler RC, Andrews G, Colpe LJ, Hiripi E, Mroczek DK, Normand S-L (2002). Short screening scales to monitor population prevalences and trends in non-specific psychological distress. Psychol Med.

[49] Sharp R (2015). The Hamilton rating scale for depression. Occup Med.

[50] Abera RG, Demesse ES, Boko WD (2022). Evaluation of glycemic control and related factors among outpatients with type 2 diabetes at Tikur Anbessa Specialized Hospital, Addis Ababa, Ethiopia: a cross-sectional study. BMC Endocr disorders.

[51] Alemu T, Tadesse T, Amogne G (2021). Glycemic control and its determinants among patients with type 2 diabetes mellitus at Menelik II Referral Hospital, Ethiopia. SAGE open medicine.

[52] Fasil A, Biadgo B, Abebe M. Glycemic control and diabetes complications among diabetes mellitus patients attending at University of Gondar Hospital, Northwest Ethiopia. Diabetes, metabolic syndrome and obesity: targets and therapy. 2019;12:75. 10.2147/DMSO.S185614 PMC6306061 30613158

[53] Fekadu G, Bula K, Bayisa G, Turi E, Tolossa T, Kasaye HK (2019). Challenges and factors associated with poor glycemic control among type 2 diabetes mellitus patients at Nekemte Referral Hospital, Western Ethiopia. J multidisciplinary Healthc.

[54] Mamo Y, Bekele F, Nigussie T, Zewudie A (2019). Determinants of poor glycemic control among adult patients with type 2 diabetes mellitus in Jimma University Medical Center, Jimma zone, south west Ethiopia: a case control study. BMC Endocr disorders.

[55] Tekalegn Y, Addissie A, Kebede T, Ayele W (2018). Magnitude of glycemic control and its associated factors among patients with type 2 diabetes at Tikur Anbessa Specialized Hospital, Addis Ababa, Ethiopia. PLoS ONE.

[56] Al-Ghamdi AA (2004). A high prevalence of depression among diabetic patients at a teaching hospital in western Saudi Arabia. Neurosciences J.

[57] Azmiardi A, Murti B, Febrinasari RP, Tamtomo DG (2022). Low social support and risk for depression in people with type 2 diabetes Mellitus: a systematic review and Meta-analysis. J Prev Med Public Health.

[58] Lin C, Lee Y, Liu C, Chen H, Ko M, Li C (2012). Urbanization and prevalence of depression in diabetes. Public Health.

[59] Dehesh T, Dehesh P, Shojaei S. Prevalence and associated factors of anxiety and depression among patients with Type 2 diabetes in Kerman, Southern Iran. Diabetes, Metabolic Syndrome and Obesity: Targets and Therapy. 2020;13:1509. 10.2147/DMSO.S249385 PMC7211308 32440180

[60] Liu X, Li Y, Guan L, He X, Zhang H, Zhang J et al. A Systematic Review and Meta-Analysis of the Prevalence and Risk Factors of Depression in Type 2 Diabetes Patients in China.Frontiers in Medicine. 2022;9. 10.3389/fmed.2022.759499 PMC9127805 35620713

[61] Kuehner C (2003). Gender differences in unipolar depression: an update of epidemiological findings and possible explanations. Acta psychiatrica Scandinavica.

[62] Kuehner C (2017). Why is depression more common among women than among men?. The Lancet Psychiatry.

[63] Bot M, Pouwer F, De Jonge P, Tack C, Geelhoed-Duijvestijn P, Snoek FJ (2013). Differential associations between depressive symptoms and glycaemic control in outpatients with diabetes. Diabet Med.

[64] Hussain S, Habib A, Singh A, Akhtar M, Najmi AK (2018). Prevalence of depression among type 2 diabetes mellitus patients in India: a meta-analysis. Psychiatry Res.

[65] Khaledi M, Haghighatdoost F, Feizi A, Aminorroaya A (2019). The prevalence of comorbid depression in patients with type 2 diabetes: an updated systematic review and meta-analysis on huge number of observational studies. Acta Diabetol.

[66] Pashaki MS, Mezel JA, Mokhtari Z, Gheshlagh RG, Hesabi PS, Nematifard T et al. The prevalence of comorbid depression in patients with diabetes: a meta-analysis of observational studies. Diabetes & Metabolic Syndrome: Clinical Research & Reviews. 2019;13(6):3113-9. 10.1016/j.dsx.2019.11.003 31790965

[67] Wang B, Yuan J, Yao Q, Li L, Yan N, Song R (2016). Prevalence and independent risk factors of depression in chinese patients with type 2 diabetes: a systematic review and meta-analysis. The Lancet Diabetes & Endocrinology.

[68] Bener A, Al-Hamaq OAA, E Dafeeah A. E. High prevalence of depression, anxiety and stress symptoms among diabetes mellitus patients.The Open Psychiatry Journal. 2011;5(1).

[69] Katon W, Von Korff M, Ciechanowski P, Russo J, Lin E, Simon G (2004). Behavioral and clinical factors associated with depression among individuals with diabetes. Diabetes Care.

[70] Almeida OP, McCaul K, Hankey GJ, Yeap BB, Golledge J, Norman PE (2016). Duration of diabetes and its association with depression in later life: the Health in Men Study (HIMS). Maturitas.

[71] Pan Q, Li Q, Deng W, Zhao D, Qi L, Huang W (2018). Prevalence of and risk factors for peripheral neuropathy in chinese patients with diabetes: a multicenter cross-sectional study. Front Endocrinol.

[72] Teliti M, Cogni G, Sacchi L, Dagliati A, Marini S, Tibollo V (2018). Risk factors for the development of micro-vascular complications of type 2 diabetes in a single-centre cohort of patients. Diabetes and Vascular Disease Research.

[73] Reis JP, Allen NB, Bancks MP, Carr JJ, Lewis CE, Lima JA (2018). Duration of diabetes and prediabetes during adulthood and subclinical atherosclerosis and cardiac dysfunction in middle age: the CARDIA study. Diabetes Care.

[74] Jing X, Chen J, Dong Y, Han D, Zhao H, Wang X (2018). Related factors of quality of life of type 2 diabetes patients: a systematic review and meta-analysis. Health Qual Life Outcomes.

[75] Diress G, Endalifer ML, Addisu A, Mengist B (2022). Association between social supports and depression among patients with diabetes mellitus in Ethiopia: a systematic review and meta-analysis. BMJ open.

[76] Ioannou M, Kassianos AP, Symeou M. Coping with depressive symptoms in young adults: Perceived social support protects against depressive symptoms only under moderate levels of stress.Frontiers in psychology. 2019:2780. 10.3389/fpsyg.2018.02780 PMC6340372 30692958

[77] Camara M, Bacigalupe G, Padilla P (2017). The role of social support in adolescents: are you helping me or stressing me out?. Int J Adolescence Youth.

[78] Leggatt M, Woodhead G (2016). Family peer support work in an early intervention youth mental health service. Early Interv Psychiat.

