# Supplementary material for: Depression and determinants among diabetes mellitus patients in Ethiopia, a systematic review and meta-analysis
Source: BMC Psychiatry. 2023 Mar 29;23:209. doi: 10.1186/s12888-023-04655-6 (PMC10052826; doi:10.1186/s12888-023-04655-6)
Supplement: Supplementary file 2 — Supplementary Material 2 Table: Quality assessment for the included Studies [file 12888_2023_4655_MOESM2_ESM.docx]

S 2 Table. Quality assessment for the included Studies

| Item | The criteria for inclusion in the sample clearly defined | Describe study setting and participant | Valid and reliable exposure measurement | Objective and standard criteria for measurement | Identified confounder | Strategies to deal with confounders | Valid and reliable outcome measurement | Appropriate statically analysis | No of ‘yes’ ‘ |
| --- | --- | --- | --- | --- | --- | --- | --- | --- | --- |
| Adane A. et al | Yes | Yes | No | Yes | Yes | Yes | Yes | Yes | 7/8=87.5 |
| Anteneh M. et al | Yes | Yes | Yes | Yes | No | No | Yes | Yes | 6/8=75 |
| Bereket B. et al | Yes | Yes | No | Yes | Yes | Yes | Yes | Yes | 7/8=8.75 |
| Biruk S. et al | Yes | Yes | No | Yes | Yes | Yes | No | Yes | 6/8=75 |
| Bonsa A. et al | Yes | Yes | No | Yes | Yes | Yes | Yes | Yes | 7/8=87.5 |
| Mengistu E. et al | Yes | Yes | Yes | Yes | Yes | No | Yes | Yes | 7/8=87.5 |
| Gedion A. et al | Yes | Yes | Yes | Yes | No | No | Yes | Yes | 6/8=75 |
| Mogessie N. et al | Yes | Yes | Yes | Yes | Yes | Yes | Yes | No | 7/8=87.5 |

| Mohammedamin H. et al | Yes | Yes | Yes | Yes | Yes | Yes | Yes | No | 7/8=87.5 |
| --- | --- | --- | --- | --- | --- | --- | --- | --- | --- |
| Mohammed E. et al | Yes | yes | No | Yes | Yes | Yes | Yes | Yes | 7/8=87.5 |
| Nigus A. et al | Yes | Yes | Yes | No | Yes | Yes | Yes | Yes | 7/8=87.5 |
| Sisay D. et al | Yes | Yes | Yes | Yes | Yes | Yes | Yes | No | 7/8=87.5 |
| Tesfa D. et al | Yes | Yes | Yes | Yes | Yes | Yes | No | Yes | 7/8=87.5 |
| Teshager W. et al | Yes | Yes | No | Yes | Yes | Yes | Yes | Yes | 7/8=87.5 |
| Tilahun B. et al | Yes | Yes | Yes | Yes | Yes | No | Yes | No | 6 /8=75 |
| Tiki/2017 | Yes | Yes | No | Yes | Yes | No | Yes | Yes | 6/8=75 |
