# Supplementary material for: Depression and determinants among diabetes mellitus patients in Ethiopia, a systematic review and meta-analysis
Source: BMC Psychiatry. 2023 Mar 29;23:209. doi: 10.1186/s12888-023-04655-6 (PMC10052826; doi:10.1186/s12888-023-04655-6)
Supplement: Supplementary file 3 — Supplementary Material 3 Table: Risk of bias of assessment for the cross-sectional studies [file 12888_2023_4655_MOESM3_ESM.docx]

S 3 Table. Risk of bias of assessment for the cross-sectional studies

| Item | External validity | | | | Internal validity | | | | | |  | |
| --- | --- | --- | --- | --- | --- | --- | --- | --- | --- | --- | --- | --- |
|  | Representativeness s of the target population | Representativeness s of the sampling frame | Radom samplin g or census | Minimal responses e bias | Data were collect d directly | Acceptable e case definition used in the study | Valid and reliable measurement t | The same mode of data collection n for all study subject | Appropriate e length of prevalence period for parameter of interest | Appropriate numerators and denominator s of interest | No of yes | **Summ ary of risk of bias** |
| Adane A. et al | Yes | Yes | No | Yes | Yes | No | Yes | Yes | Yes | Yes | 8 | Low-  risk |
| Anteneh M. et al | Yes | Yes | Yes | Yes | No | No | Yes | Yes | Yes | Yes | 8 | Loiw risk |
| Bereket B. et al | Yes | Yes | No | Yes | Yes | No | Yes | Yes | Yes | Yes | 8 | Low- risk |
| Biruk S. et al | Yes | Yes | No | Yes | No | Yes | Yes | Yes | No | Yes | 7 | Moderate – risk |
| Bonsa A. et al | Yes | Yes | No | Yes | Yes | Yes | Yes | Yes | Yes | Yes | 9 | Low- risk |
| Mengistu E. et al | Yes | Yes | Yes | Yes | Yes | No | Yes | Yes | Yes | Yes | 9 | Low- risk |
| Gedion A. et al | Yes | Yes | No | Yes | Yes | Yes | Yes | Yes | Yes | Yes | 9 | Low- risk |
| Mogessie N. et al | Yes | Yes | Yes | Yes | No | No | Yes | Yes | Yes | Yes | 8 | Low- risk |
| Mohammedamin H. et al | Yes | Yes | No | Yes | Yes | Yes | Yes | No | Yes | Yes | 8 | Low- risk |

| Mohammed E. et al | Yes | Yes | Yes | Yes | No | Yes | Yes | No | Yes | Yes | 8 | Low- risk |
| --- | --- | --- | --- | --- | --- | --- | --- | --- | --- | --- | --- | --- |
| Nigus A. et al | Yes | Yes | Yes | Yes | No | No | Yes | Yes | Yes | Yes | 8 | Low-  risk |
| Sisay D. et al | Yes | yes | No | Yes | Yes | No | Yes | Yes | Yes | Yes | 8 | Low- risk |
| Tesfa D. et al | Yes | Yes | Yes | No | Yes | No | Yes | Yes | Yes | Yes | 8 | Low-  risk |
| Teshager W. et al | Yes | Yes | Yes | Yes | Yes | Yes | Yes | No | Yes | Yes | 9 | Low-  risk |
| Tilahun B. et al | Yes | Yes | Yes | Yes | Yes | Yes | No | No | Yes | Yes | 7 | Moderate- risk |
| Tiki/2017 | Yes | Yes | No | Yes | Yes | Yes | Yes | Yes | Yes | Yes | 9 | Low risk |
