# Supplementary figures and images for: Depression and determinants among diabetes mellitus patients in Ethiopia, a systematic review and meta-analysis
Source: BMC Psychiatry. 2023 Mar 29;23:209. doi: 10.1186/s12888-023-04655-6 (PMC10052826; doi:10.1186/s12888-023-04655-6)

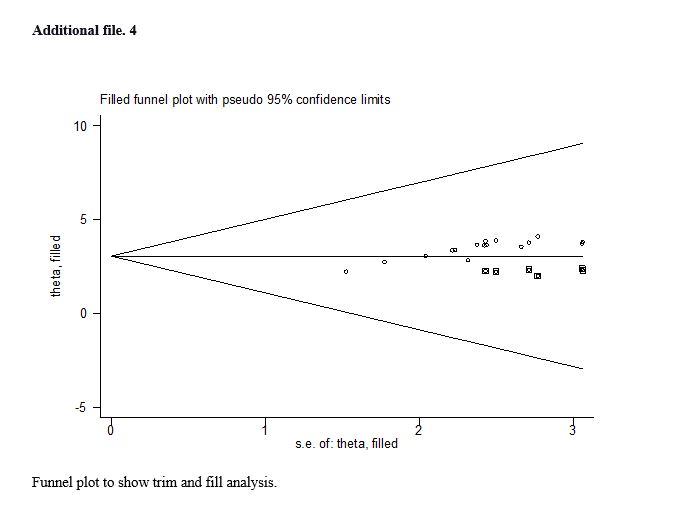

Supplement: Supplementary file 4 — Supplementary Material 4 Funnel plot to show trim and fill analysis [file 12888_2023_4655_MOESM4_ESM.jpg]
