# Supplementary material for: Depression and determinants among diabetes mellitus patients in Ethiopia, a systematic review and meta-analysis
Source: BMC Psychiatry. 2023 Mar 29;23:209. doi: 10.1186/s12888-023-04655-6 (PMC10052826; doi:10.1186/s12888-023-04655-6)
Supplement: Supplementary file 5 — Supplementary Material 5 Legends [file 12888_2023_4655_MOESM5_ESM.docx]

**Legends**

**Additional file 1 -** PRISMA checklist.

**Additional file 2 -** Methodological quality assessment of included studies using Joanna Brigg's Institute quality appraisal criteria scale (JBI). The eight item questions assessing inclusion criteria, study setting and participant, exposure measurement, objectives, confounder, statically analysis, outcome measurement and dealing confounder were used.

**Additional file 3 ­-** Risk of bias assessment for the included studies. The ten item questions of which four items assess external and six items assess internal validity were used.

**Additional file 4 –** Funnel plot to show trim and fill analysis.

**Fig 1.** PRISMA flow chart illustrating the process of search and selection of studies included in the present systematic review and meta-analysis.

**Fig 2.** The pooled prevalence of depression among diabetes in Ethiopia.

**Fig 3.** Funnel plot which shows the asymmetric distribution of studies.

**Fig 4.** Results of sensitivity analysis of the 16 studies in the meta-analysis of depression among diabetes.
